# The Effect of Detraining after a Period of Training on Cardiometabolic Health in Previously Sedentary Individuals

**DOI:** 10.3390/ijerph15102303

**Published:** 2018-10-19

**Authors:** Paul B. Nolan, Shawn M. Keeling, Chantelle A. Robitaille, Christina A. Buchanan, Lance C. Dalleck

**Affiliations:** 1 College of Nursing and Health Sciences, Flinders University, Adelaide 5001, Australia; 2 Recreation, Exercise & Sports Science Department, Western State Colorado University, Gunnison, CO 81231, USA; skeeling@western.edu (S.M.K.); Chantelle.robitaille@outlook.com (C.A.R.); chbuchanan@western.edu (C.A.B.); ldalleck@western.edu (L.C.D.)

**Keywords:** physical inactivity, metabolic syndrome, cardiorespiratory fitness, resistance training, aerobic training

## Abstract

The purpose of this study was to quantify the time-magnitude changes in cardiometabolic health outcomes that occur with cessation of regular exercise training. All participants (*n* = 22) performed baseline testing, completed a 13-week exercise program, and completed post-program testing. Upon completion of the 13-week exercise program, participants were randomized to one of the following two treatment groups: (1) the treatment group that continued their exercise for 4 weeks (TRAIN); or (2) the treatment group that discontinued exercise (DETRAIN). Changes from baseline to 13 weeks in both the TRAIN and DETRAIN treatment groups for maximal oxygen consumption (VO_2_max), body fat percentage, mean arterial pressure, high-density lipoprotein (HDL) cholesterol, and triglycerides were significantly favourable (*p* < 0.05). VO_2_max, body fat percentage, and favourable cardiometabolic health adaptations continued to improve (*p* < 0.05) with an additional one month of exercise training. Upon cessation of exercise, all measures of VO_2_max and body fat percentage, along with mean arterial pressure, HDL cholesterol, and triglycerides significantly worsened (*p* < 0.05) in the DETRAIN treatment group. Favourable training adaptations were further enhanced with an additional month of continued exercise training, and cessation of regular exercise rapidly abolished all training adaptations within one month. These novel findings underscore the importance of sustained and uninterrupted exercise training.

## 1. Introduction

There is a robust inverse relationship between cardiorespiratory fitness (CRF) and risk of mortality from cardiovascular disease (CVD) and all causes [1]. For example, a one metabolic equivalent (1-MET) increase in CRF in low-risk middle aged men and women is reported to promote an 18% reduction in CVD mortality [2]. Therefore, increasing CRF via exercise training and increased physical activity (PA) is a pertinent public health prevention measure to improve CVD-free survival in low risk adults.

Findings from numerous epidemiological studies clearly demonstrate that physical inactivity is associated with a higher prevalence of most CVD risk factors, including abnormal lipids, high blood pressure (BP), metabolic syndrome (MetS), obesity, and type 2 diabetes [3]. Furthermore, physical inactivity is linked to most prevalent chronic diseases and is estimated to contribute to approximately 250,000 premature deaths annually, representing approximately one quarter of all preventable deaths [4].

However, the majority of research linking either CRF or physical inactivity to disease risk or cardiometabolic risk factors has focused on data collected at various time points with little standardization of the days or weeks prior to data collection [5]. Furthermore, most research has collected follow-up data in the 48–72 h following the last exercise bout [6]. While this research has been valuable in promoting exercise in the public health domain, it is still unclear what the effects of intermittent periods of increased activity or inactivity are on improvements in cardiometabolic health following standardized aerobic exercise training periods of 12–24 weeks [6,7]. This is problematic, as periodic activity or inactivity more closely mimics the fluctuation in activity levels in individuals as they go about their daily lives. Common issues such as low initial fitness, low motivation, seasonal holidays, travel, injury, and illness have all been cited [6] as reasons for low adherence or cessation of exercise training. Thus evaluation of the effect of stopping an exercise intervention on cardiometabolic health in apparently healthy adults is an important area to understand.

Accordingly, the purpose of this study was to examine the cardiometabolic health implications with detraining after a regular exercise-training program. The main aim was to quantify the time-magnitude changes in CRF and cardiometabolic health outcomes that occur with cessation of regular exercise training. It was hypothesized that CRF and cardiometabolic health will decline rapidly with the absence of regular exercise training over a one-month timeframe.

## 2. Materials and Methods

Thirty-five non-smoking men and women (aged 22 to 77 years) were recruited into the study if they were of low-to-moderate risk as defined by the American College of Sports Medicine [7] and not physically active (not participating in at least 30 min of moderate intensity physical activity on at least three days of the week for at least three months). Participants were also eligible for inclusion into the study if they verbally agreed to continue previous dietary habits and not perform additional exercise beyond that required for the present study. Exclusionary criteria included evidence of CVD, or pulmonary, and/or metabolic disease as determined by medical history questionnaire. This study was approved by the Human Research Committee at Western State Colorado University (HRC2017-01-01R20). Each participant signed an informed consent form prior to participation.

**Figure 1:**
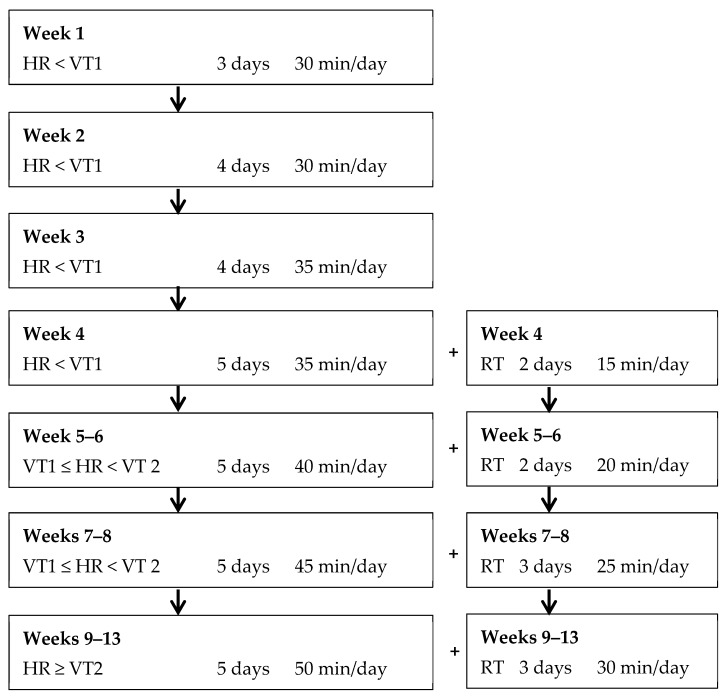
Week-to-week exercise prescription for days/times of cardiorespiratory (left-hand side) and resistance training (RT) (right-hand side). HR—heart rate, VT—ventilatory threshold.

All participants performed baseline testing as outlined below and completed an individualized 13-week exercise program (Figure 1) according to the American Council of Exercise (ACE) Integrated Fitness Training (IFT) model guidelines [8], and completed post-program testing. Upon completion of the 13-week exercise training program and post-program testing participants were randomized to either of the following two treatment groups: The continued training group (TRAIN, *n* = 17) continued their individualized exercise program according to the ACE IFT model guidelines for an additional four weeks and; the ceased treatment group (DETRAIN, *n* = 18) discontinued regular exercise. Participants in DETRAIN did not perform any structured exercise whatsoever for four weeks; however, they were permitted to maintain other lifestyle habits (e.g., nutrition and activities of daily living).

All variables were measured at the initial assessment and following the 13 weeks of exercise training. In the detraining period, CRF and skinfold assessment occurred at weeks two and four only. Measures of BP, blood lipids, fasting blood glucose, waist circumference, and weight were obtained in each of the four weeks during the post-exercise training period.

Participants completed a maximum graded exercise test (GXT) on a motorized treadmill (Powerjog GX200, Inspire Fitness Solutions Ltd., Biddeford, ME, USA). Participants walked or jogged at a self-selected pace before the treadmill incline was increased by 1% every minute until the participant reached volitional fatigue. Participants heart rate (HR) were continuously recorded via a chest strap and radio-telemetric receiver (Polar Electro, Woodbury, NY, USA). Expired air and gas exchange data were recorded continuously using a metabolic analyser (Parvo Medics TrueOne 2.0, Parvo Medics Inc., Salt Lake City, UT, USA). Before each exercise test, the metabolic analyser was calibrated with gases of known concentrations (14.01 ± 0.07% O_2_, 6.00 ± 0.03% CO_2_) and with room air (20.93% O_2_ and 0.03% CO_2_) as per the instruction manual. Volume calibration of the pneumotachometer was done via a 3-litre calibration syringe system (Hans-Rudolph, Kansas City, MO, USA). The last 15 s of the GXT were averaged—This was considered the final data point. The closest neighbouring data point was calculated by averaging the data collected 15 s immediately before the last 15 s of the test. The mean of the two processed data points represented the VO_2_max. The criteria for attainment of maximal oxygen consumption (VO_2_max) were two out of three of the following: (1) a plateau (∆VO_2_ ≤ 150 mL/min) in VO_2_ with increases in workload; (2) maximal respiratory exchange ratio (RER) ≥ 1.1; and (3) maximal HR within 15 beats/min of the age-predicted maximum (220–age).

Determination of both the first ventilatory threshold (VT1) and second ventilatory threshold (VT2) were made by visual inspection of graphs of time plotted against each relevant respiratory variable (according to 15 s time-averaging). The criteria for VT1 was an increase in VE/VO_2_ with no concurrent increase in VE/VCO_2_ and departure from the linearity of VE. The criteria for VT2 was a simultaneous increase in both VE/VO_2_ and VE/VCO_2_. The corresponding HRs at VT1 and VT2 were used to improve the robustness of the exercise training response as has been shown elsewhere [1]. All analysis to determine the VTs were done independently by two experienced exercise physiologists. In the event of conflicting results, the original assessments were re-evaluated and collectively a consensus was agreed upon.

Participants were weighed to the nearest 0.1 kg on a medical grade scale and measured for height to the nearest 0.5 cm using a stadiometer. Percent body fat (FAT) was determined via skinfolds [7]. Skinfold thickness was measured to the nearest ±0.5 mm using a Lange calliper. All measurements were taken on the right side of the body using standardized anatomical sites (three-site) for men (chest, abdomen, thigh) and women (tricep, suprailiac, thigh). These measurements were performed until two were within 10% of each other. All skinfold measures were obtained by the same qualified clinical exercise physiologist. Waist circumference (WC) measurements were obtained using a cloth tape measure with a spring loaded-handle. A horizontal measurement was taken at the narrowest point of the torso (below the xiphoid process and above the umbilicus). These measurements were taken until the two were within 0.5 mm of each other.

A fasting blood sample was collected and analysed for measurement of lipids and glucose. A fingerstick sample was collected into heparin-coated 40-μL capillary tube. Blood flowed freely from the fingerstick into the capillary tube without milking of the finger. Samples were dispensed immediately onto commercially available test cassettes for analysis in a Cholestech LDX System (Abbott Ltd., Chicago, IL, USA) according to strict standardized operating procedures.

The procedures for assessment of resting HR and BP outlined elsewhere were followed [7] and collected in a standardized manner. The mean of the two measurements was reported for baseline and post-program values.

### 2.1. Cardiometabolic Health

Cardiometabolic risk was determined via calculation of a MetS z-score following the procedure outlined and used elsewhere. Briefly, this score is the sum of the participant’s MetS components relative to the threshold for determination of each component. The MetS z-score has been used previously to identify changes in MetS risk factors following an exercise intervention [9,10]. The sex-specific MetS z-scores were calculated using the following equations [9,10]: (1) MetS z-score_men_ = [(40 − HDL)/8.9] + [(TG − 150/69)] + [(FG − 100)/17.8] + [(WC − 102)/11.5] + [(MAP − 100)/10.1]; (2) MetS z-score_women_ = [(50 − HDL)/14.5] + [(TG − 150/69)] + [(FG − 100)/17.8] + [(WC − 88)/12.5] + [(MAP − 100)/10.1], where FG = fasting glucose; HDL = high-density lipoprotein cholesterol; MAP = mean arterial pressure; TG = triglycerides; and WC = waist circumference.

### 2.2. Exercise Prescription

All exercise was supervised one-to-one by student-trainers under the supervision of an experienced researcher. No specific motivation strategies were employed, and participants were booked in for their training at individual times according to their preferences and availability. Exercise training was progressed according to recommendations made elsewhere by ACE [8] and implemented in previous research [11,12]. Polar HR monitors were used to monitor HR during all exercise sessions. Researchers adjusted workloads on aerobic modalities accordingly during each exercise session to ensure actual HR responses aligned with target HR. The week-to-week exercise prescription for cardiorespiratory and resistance training (RT) modes are provided in Figure 1 for the 13-week exercise programme.

### 2.3. Statistical Analyses

Measures of centrality and spread are presented as mean ± SD. Paired *t*-tests were used to determine mean within-group differences between baseline and week 13 for all physical fitness and cardiometabolic health outcome measurements. Independent *t*-tests were performed to compare treatment group (TRAIN vs. DETRAIN) at baseline and week 13 for all physical fitness and cardiometabolic health outcome measurements.

#### Post-Training Period

Repeated measures ANOVA were performed for the TRAIN and DETRAIN groups for all variables listed in Table 1 with the exception of height and age. Repeated measures were used to compare differences in the change (from baseline) at the end of training, and each week of the post-training period for CRF, FAT, MetS z-score, HDL, TG, BG, MAP, and WC. Unpaired *t*-tests were performed to determine group differences between TRAIN and DETRAIN for each time point in the post-training period. One-way ANOVA was performed to determine the main effect of time for the TRAIN and DETRAIN groups during the post-training period. Pairwise comparison analyses were utilized to determine where significant differences existed between each time point.

Statistical significance was set at α = 0.05 for all analyses. All analyses were performed using SPSS Version 25.0 (IBM, Chicago, IL, USA) and GraphPad Prism 7.0 (GraphPad Software Inc., San Diego, CA, USA).

## 3. Results

All analyses and data presented in the results are for those participants who completed the investigation and full data sets were available. Six participants were unable to complete the initial 13-week training period for the following reasons: personal reasons (*n* = 3), illness (*n* = 2), and out-of-town move (*n* = 1). The dropout rate was greater in the TRAIN group (*n* = 4) vs. DETRAIN group (*n* = 2). No adverse events due to the exercise training were reported in either group. A further seven participants did not complete all testing sessions during the four week timeframe of continued training or detraining and were therefore excluded from the analysis.

Overall, there was excellent compliance in both groups to the total number of prescribed training sessions for the initial 13 weeks of exercise training: TRAIN group—mean, 88.7% (range, 77.4–96.8%) and DETRAIN group—mean, 90.6% (range, 80.6–100.0%). Compliance in the TRAIN group for the four weeks of continued exercise training was also excellent: mean, 91.3% (range, 80.0–100.0%).

The anthropometric, CRF, MetS Z-score, and individual cardiometabolic measures at baseline and 13 weeks for participants in both treatment groups are shown in Table 1.

After the initial 13 weeks of exercise training, there were significant changes in body fat percentage, VO_2_max, HDL cholesterol, and MetS z-score in both groups. Systolic BP, total cholesterol, and TG values were significantly different after 13 weeks in the TRAIN group only, whereas LDL cholesterol was significantly different in the DETRAIN group only (all *p* > 0.05). After 13 weeks, the TRAIN and DETRAIN treatment groups were only statistically significantly different in MetS z-score (*p* = 0.032).

### 3.1. Maintain Regular Exercise Training

Anthropometric, cardiometabolic risk, and CRF measures at post-program and throughout the one-month timeframe of continued exercise for the TRAIN group are shown in Table 2. CRF and body fat percentage continued to improve (*p* < 0.05) with an additional one month of sustained individualized exercise training. Moreover, after one month of continued regular exercise training, the favourable adaptations in systolic BP, HDL cholesterol, and TG observed during the initial 13 weeks of exercise continued to be sustained (*p* > 0.05) although there was no further improvement in MetS z-score (*p* > 0.05). Similar to the initial 13-week training block, all other measures (weight, WC, diastolic BP, total cholesterol, LDL cholesterol, and BG) remained unchanged (*p* > 0.05) despite an additional one month of exercise.

### 3.2. Detrain from Exercise Training

Anthropometric, cardiometabolic risk, and CRF measures at post-program and throughout the one-month timeframe of continued exercise for the DETRAIN group are shown in Table 3. Upon cessation of exercise training, VO_2_max, body fat percentage, HDL, TG, and MetS z-score significantly worsened (*p* < 0.05). Weight, WC, systolic and diastolic BP, total cholesterol, LDL cholesterol, and BG were unchanged (*p* > 0.05) during the one month follow-up period of physical inactivity.

### 3.3. Change in Cardiometabolic Health in the Post-Training Period

**Figure 2:**
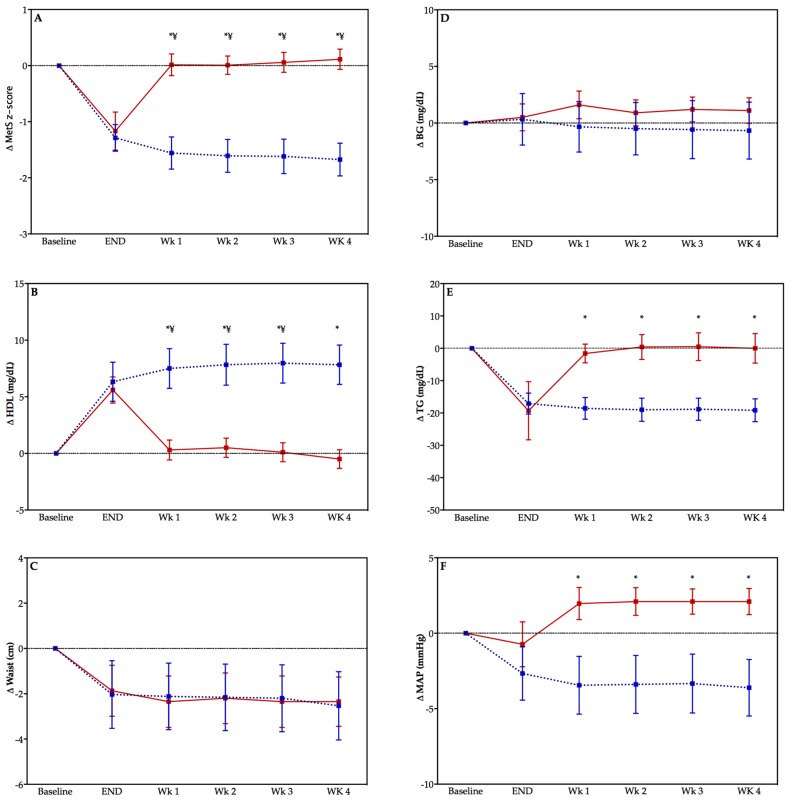
Change in MetS z-score (panel **A**) and the individual components of MetS z-score (panels **B**–**F**) in previously sedentary adults who either detrained (DETRAIN; red bars, solid line), or trained (TRAIN; blue bars, dotted lines). Please note that baseline is not included in statistical analysis but included to indicate the change in the variable with the exercise training program. Data displayed as mean ± S.E.M, * *p* < 0.05 comparison between groups, ^¥^ *p* < 0.05 compared with “end” time point DETRAIN group. MetS: metabolic syndrome; BG: fasting blood glucose; HDL: high-density lipoprotein; TG: triglycerides; MAP: mean arterial pressure.

There were significant differences between the TRAIN and DETRAIN groups at each week in the detraining period for change in MetS z-score (all *p* < 0.05)—see Figure 2 (panel A). Significant differences exist between groups in HDL cholesterol (panel C), TG (panel D), and MAP (panel F) but not WC (panel E), or BG (panel B). There was a significant effect of time on MetS z-score (panel A) and HDL (panel C) only (*p* < 0.05).

### 3.4. Change in CRF and Body Fat Percentage in the Post-Training Period

**Figure 3:**
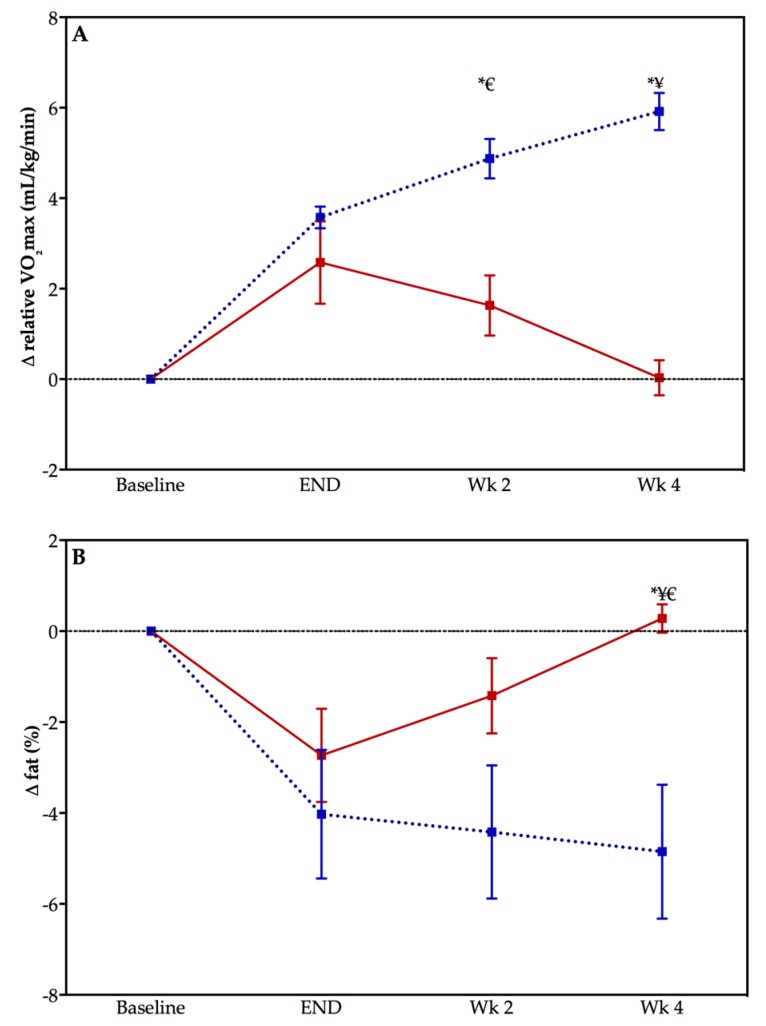
Change in cardiorespiratory fitness (panel **A**) and body fat percentage (panel **B**) in previously sedentary adults who either detrained (DETRAIN; red bars, solid line), or trained (TRAIN; blue bars, dotted line). Please note that baseline is not included in statistical analysis but is included to indicate the change in the variable with the exercise training program. Data displayed as mean ± S.E.M, * *p* < 0.05 comparison between groups, ^¥^ *p* < 0.05 compared with “end” time point DETRAIN group, ^€^ *p* < 0.05 compared with “end” time point TRAIN group.

Figure 3 displays the graphs of change in CRF (panel A) and body fat percentage (panel B) during the post-training period for both the TRAIN and DETRAIN groups. There were significant differences in the change in CRF between the TRAIN and DETRAIN groups at both week 2 and week 4 in the detraining period (both *p* < 0.005). The change in body fat percentage was only different between groups at week 4 (*p* < 0.005). The main effect of time was significantly different at both week 2 (*p* = 0.015) and week 4 (*p* < 0.005) for change in CRF from end of training but only for week 4 (*p* = 0.038) for the change in body fat percentage for the TRAIN group. The main effect of time was significantly different at week 4 for change in CRF (*p* = 0.022) from end of training and for the change in body fat percentage for the DETRAIN group (*p* = 0.033).

## 4. Discussion

The major finding of this study is the improvements in cardiometabolic health following 13 weeks of exercise training reverted with one week of detraining. Secondary analyses of the relationship of individual MetS components that contribute to cardiometabolic health reveal the deterioration with detraining manifests chiefly due to a reduction in HDL cholesterol, an increase in TG concentration, and a rise in MAP above the initial baseline level. Lastly, cessation of regular exercise rapidly abolished all training adaptations with one month of detraining. These novel findings underscore the importance of sustained and uninterrupted exercise training for improving cardiometabolic health and primary prevention of chronic disease.

### 4.1. Primary Prevention of Chronic Disease Perspective

In the past few decades low CRF has garnered considerable attention as an independent and powerful predictor of CVD risk and premature mortality. For instance, Williams (2001) showed in a meta-analysis that there was a marked decrease in relative risk for CVD when individuals moved out of the lowest quartile of CRF [13]. Blair (2009) estimated that low CRF accounted for more overall deaths when compared to deaths which could be attributed to traditional CVD risk factors, such as obesity, smoking, hypertension, high cholesterol, and diabetes [14]. Accordingly, the changes in CRF, as indicated by the measured VO_2_max, have clinical and public health relevance, as a large number of adults fall into clinically-defined low CRF categories and therefore demonstrate increased CVD risk [15]. Overall, VO_2_max improved on average by 1.69 METs following 17 weeks (1.03 METs at 13 weeks) of exercise training in the TRAIN treatment group. These improvements likely have important long-term prevention implications as a recent study reported a 1 MET increase in VO_2_max was associated with an 18% reduction in deaths due to CVD [2]. However, the initial improvement in VO_2_max (0.7 MET at 13 weeks) and likely reduced risk of CVD experienced in the DETRAIN treatment group was rapidly lost upon 4-week cessation of exercise training (total change 0.02 METs).

A classic study from the early 1990s demonstrated the insidious outcomes from even small interruptions to regular physical activity [16]. The researchers had a group of men perform 10 weeks of single leg cycle ergometry at an intensity of 70% of their VO_2_max. This experimental design allowed the untrained leg to serve as the control. After 10 weeks of training there was a 25% improvement in insulin sensitivity in the trained leg, which corresponds to better blood glucose control. Remarkably, all of the favourable training adaptations were lost after only six days without exercise.

Although there is almost universal agreement in the literature that various cardiometabolic health indicators decline with the cessation of training [16,17,18,19], there is considerable variability reported on which parameters change during the detraining period. Bajpeyi et al. (2009) reported improvements in insulin sensitivity with eight months exercise training but different responses in insulin sensitivity due to detraining depending on the exercise volume and intensity applied during the training period [17]. Similar to the current study, Nikseresht et al. (2016) reported a decreased HDL cholesterol with detraining in middle aged obese men following 12 weeks of aerobic interval training (AIT), although other blood lipid parameters did not show a decline with detraining [18]. Similarly, after four months of AIT, Mora-Rodriguez et al. (2014) reported no change in WC, and a decline in HDL cholesterol and CRF after one month of detraining [19]. However, Mora-Rodriguez reported the improvements in BP with training were maintained in the detraining period in contrast to the current study [19].

While there is some discrepancy in the various cardiometabolic risk parameters, all studies including the current study report a rapid decline (within days to weeks) in some of them with the cessation of training. Decline in cardiometabolic health has been reported as occurring from six days [16] to eight weeks [20] although it should be pointed out that these timelines are somewhat arbitrary in that most studies have not looked at shorter or longer timeframes for data collection. Unique to the current study however, the changes we saw in cardiometabolic health were most prominent in the first week with a small degradation in the subsequent three weeks. This likely indicates that in previous sedentary individuals, many of the health benefits associated with short-term exercise training are lost rapidly and emphasis on longer-term training is required for the realization of the primary prevention benefits of starting exercise. Collectively these findings point to the importance of maintaining a persistent frequency of exercise training to maintain the cardiometabolic health benefits related to exercise. Determining the minimum frequency, intensity, duration, and volume of exercise to maintain the health benefits remains to be determined and represents important future work in the area of exercise and cardiometabolic health.

### 4.2. Methodological Considerations

A few limitations to the present study warrant further discussion. First, while participants were instructed to maintain their regular dietary intake during the 13-week intervention, diet intake was not strictly controlled for in this study. Second, physical activity/sedentary behaviour outside of the training program and prescribed medications were not monitored, and thus may have influenced the current findings. Third, participants were included in the study based on their sedentary behaviour history and were not matched based on their cardiometabolic health. Between-group differences do exist in some cardiometabolic health parameters that may have a small effect on the results. This limitation may be overcome in future studies with larger participant numbers to improve the generalizability of the results. Lastly, while we used a score of cardiometabolic health, apart from sedentariness, the participants in this study were apparently healthy.

## 5. Conclusions

We have demonstrated that changes in cardiometabolic health are attainable in previously sedentary individuals with short-term exercise training. However, the key finding in this study is that in those people who subsequently detrained, the cardiometabolic health benefits were nearly completely reversed within one week of detraining. This research highlights the importance of sustained and uninterrupted exercise training for the realization of the public health benefits of exercise in the prevention of chronic disease.

## Figures and Tables

**Table 1:** Anthropometric, cardiometabolic risk, and cardiorespiratory fitness data for the TRAIN and DETRAIN groups before and immediately after the 13-week exercise training programme.

Parameter	TRAIN (*n* = 12)		DETRAIN (*n* = 10)	
	Baseline	13 Weeks	Baseline	13 Weeks
**Age (years)**	40 ± 3	____	36 ± 5	____
**Height (cm)**	167.9 ± 1.9	____	169.7 ± 4.0	____
**Weight (kg)**	78.1 ± 8.6	78.9 ± 7.3	72.7 ± 16.2	72.2 ± 16.1
**Waist circumference (cm)**	86.5 ± 6.7	84.5 ± 3.6	81.7 ± 11.2	79.8 ± 10.1
**Body fat (%)**	31.1 ± 6.5	27.0 ± 2.2 *	28.5 ± 7.0	25.8 ± 9.1 *
**VO_2_max (mL·kg^−1^·min^−1^)**	30.6 ± 8.4	34.2 ± 8.7 *	33.7 ± 9.4	36.3 ± 7.4 *
**Systolic BP (mmHg)**	120 ± 4 ^¥^	117 ± 6 *	113 ± 8	108 ± 14
**Diastolic BP (mmHg)**	82 ± 10 ^¥^	80 ± 7	72 ± 8	74 ± 7
**Total cholesterol (mg·dL^−1^)**	163 ± 34	177 ± 25 *	188 ± 36	201 ± 34
**HDL cholesterol (mg·dL^−1^)**	55 ± 11	61 ± 14 *	57 ± 15	63 ± 15 *
**LDL cholesterol (mg·dL^−1^)**	89 ± 29	95 ± 19	112 ± 33	125 ± 41 *
**Triglycerides (mg·dL^−1^)**	114 ± 28	97 ± 27 *	112 ± 34	92 ± 23
**Blood glucose (mg·dL^−1^)**	92 ± 9	92 ± 11	92 ± 8	92 ± 7
**MetS z-score**	−3.8 ± 1.9 ^¥^	−5.1 ± 1.9 ^¥,^*	−5.9 ± 2.1	−7.0 ± 1.9 *

Data displayed as mean ± SD, * Within-group (TRAIN and DETRAIN) is significantly different from baseline, *p* < 0.05. ^¥^ Between-group difference (baseline and 13 weeks), *p* < 0.05. BP: blood pressure; HDL: high-density lipoprotein; LDL: low-density lipoprotein; MetS: metabolic syndrome; VO_2_max: maximal oxygen consumption.

**Table 2:** Anthropometric, cardiometabolic risk, and cardiorespiratory fitness data for participants who continued training (TRAIN) (*n* = 12) after the initial 13-week exercise training programme.

Parameter	Post Program	+1 Week	+2 Weeks	+3 Weeks	+4 Weeks
**Weight (kg)**	78.9 ± 7.3	78.6 ± 7.1	78.4 ± 7.5	78.3 ± 7.2	78.0 ± 7.2
**Waist circumference (cm)**	84.5± 3.6	84.4 ± 3.7	84.3 ± 3.7	84.3 ± 3.9	84.0 ± 3.7
**Body fat (%)**	27.1 ± 2.2	____	26.7 ± 2.4	____	26.3 ± 2.2 *^,¥^
**VO_2_max (mL·kg^−1^·min^−1^)**	34.2 ± 8.7	____	35.5 ± 8.3 *	____	36.5 ± 8.0 *^,¥^
**Systolic BP (mmHg)**	117 ± 6	116 ± 6	116 ± 6	116 ± 6	115 ± 6
**Diastolic BP (mmHg)**	80 ± 7	79 ± 6	79 ± 5	79 ± 6	79 ± 5
**Total cholesterol (mg·dL^−1^)**	177 ± 25	176 ± 23	178 ± 23	178 ± 24	180 ± 25
**HDL cholesterol (mg·dL^−1^)**	61 ± 14	63 ± 13	63 ± 13	63 ± 13	63 ± 13
**LDL cholesterol (mg·dL^−1^)**	94 ± 18	93 ± 18	94 ± 19	93 ± 17	93 ± 17
**Triglycerides (mg·dL^−1^)**	97 ± 27	96 ± 22	95 ± 21	96 ± 21	95 ± 20
**Blood glucose (mg·dL^−1^)**	92 ± 11	92 ± 9	91 ± 9	91 ± 10	91 ± 10
**MetS z-score**	−5.1 ± 1.9	−5.3 ± 1.8	−5.4 ± 1.8	−5.4 ± 1.8	−5.5 ± 1.8

Data displayed as mean ± SD, * *p* < 0.05 from post-program, ^¥^ *p* < 0.05 from +2 weeks. BP: blood pressure; HDL: high-density lipoprotein; LDL: low-density lipoprotein; MetS: metabolic syndrome; VO_2_max: maximal oxygen consumption.

**Table 3:** Anthropometric, cardiometabolic risk, and cardiorespiratory fitness data for participants that detrained (DETRAIN) (*n* = 10) after the 13-week exercise training programme.

Parameter	Post-Program	+1 Week	+2 Weeks	+3 Weeks	+4 Weeks
**Weight (kg)**	72.2 ± 16.1	72.4 ± 16.2	72.4 ± 16.5	72.6 ± 16.5	72.6 ± 16.5
**Waist circumference (cm)**	79.8 ± 10.1	79.4 ± 10.0	79.5 ± 10.1	79.4 ± 10.0	79.4 ± 9.9
**Body fat (%)**	25.8 ± 9.1	____	27.1 ± 8.5	____	28.8 ± 6.9 *
**VO_2_max (mL·kg^−1^·min^−1^)**	36.3 ± 7.4	____	35.4 ± 8.0	____	33.8 ± 8.9 *^,¥^
**Systolic BP (mmHg)**	108 ± 14	114 ± 9	115 ± 8	115 ± 8	115 ± 7
**Diastolic BP (mmHg)**	74 ± 7	75 ± 8	75 ± 8	75 ± 7	75 ± 8
**Total cholesterol (mg·dL^−1^)**	201 ± 34	200 ± 31	201 ± 31	202 ± 31	203 ± 31
**HDL cholesterol (mg·dL^−1^)**	63 ± 15	57 ± 17 *	57 ± 17 *	57 ± 17 *	57 ± 17 *
**LDL cholesterol (mg·dL^−1^)**	125 ± 41	125 ± 40	125 ± 40	125 ± 39	126 ± 40
**Triglycerides (mg·dL^−1^)**	92 ± 23	110 ± 40	112 ± 39	112 ± 37 *	112 ± 36 *
**Blood glucose (mg·dL^−1^)**	92 ± 7	93 ± 7	93 ± 7	93 ± 7	93 ± 7
**MetS z-score**	−7.0 ± 1.9	−5.8 ± 2.1 *	−5.9 ± 2.1 *	−5.9 ± 2.1 *	−5.7 ± 2.1 *

Data displayed as mean ± SD, * *p* < 0.05 from post-program, ^¥^ *p* < 0.05 from +2 weeks. BP: blood pressure; HDL: high-density lipoprotein; LDL: low-density lipoprotein; MetS: metabolic syndrome; VO_2_max: maximal oxygen consumption.
